# Medium-Term Effect of Inhaled Nitric Oxide in Mechanically Ventilated COVID-19 Patients

**DOI:** 10.3390/jcm14030806

**Published:** 2025-01-26

**Authors:** Lev Freidkin, Tamar Garsiel Katz, Ido Peles, Itamar Ben Shitrit, Marya Abayev, Yaniv Almog, Ori Galante, Lior Fuchs

**Affiliations:** 1 Pulmonary Division, Rabin Medical Center, Petah Tikva 4941492, Israel; 2 School of Medicine, Faculty of Medical and Health Sciences, Tel Aviv University, Tel Aviv 6997801, Israel; 3 Joyce and Irving Goldman Medical School, Faculty of Health Science, Ben-Gurion University of the Negev, Beer-Sheva 8410501, Israel; tamargarkatz@gmail.com (T.G.K.);; 4 Medical Intensive Care Unit, Soroka University Medical Center, Beer-Sheva 8410101, Israel; 5 Clinical Research Center, Soroka University Medical Center, Faculty of Health Sciences, Ben-Gurion University of the Negev, Beer-Sheva 8410101, Israel

**Keywords:** inhaled nitric oxide, ARDS, COVID-19

## Abstract

**Background**: Nitric oxide (NO) plays a key role in various physiological processes. Inhaled NO (iNO) has been studied for treating acute respiratory distress syndrome (ARDS). During the COVID-19 pandemic, interest grew in its potential role for patients with COVID-19 ARDS, with studies showing improved oxygenation over 48 h. **Methods**: This is a retrospective study of adult patients with severe COVID-19 ARDS and refractory hypoxemia admitted to the medical ICU requiring mechanical ventilation and treated with iNO. The effect on oxygenation, respiratory, and ventilation parameters is measured. Significant improvement is defined as an increase in PaO_2_/FiO_2_ ≥ 20% from a baseline. **Results**: This study includes 87 patients (55 men, mean age 58.7 ± 15.2) with 164 iNO connections (mean 1.9 per patient). iNO is independently associated with a significant PaO_2_/FiO_2_ ratio improvement, with an OR of 1.26 (95% CI 1.09–1.46), even after accounting for these potential confounders. The time to maximal PaO_2_/FiO_2_ improvement is 14.5 ± 5.0 h for men and 78.5 ± 5.5 h for women, with respective ratio increases of 42.5 ± 8.1 and 52.5 ± 13.6 mmHg. **Conclusions**: Our study demonstrates that severe ARDS COVID-19 patients may benefit from inhaled nitric oxide, with delayed oxygenation improvements lasting up to 96 h and slower responses observed in women, raising the possibility that current guidelines against its use could be reconsidered.

## 1. Introduction

Since the discovery of the physiological role of nitric oxide (NO) in the late 1980s, it has garnered increasing attention from clinicians for its therapeutic potential. The National Center for Biotechnology Information (NCBI) database contains over 5000 English-language articles from the past 30 years on clinical trials involving NO in humans. It has been demonstrated that nitric oxide is involved in several critical physiological processes. It facilitates vasodilation through the activation of guanylate cyclase, leading to an increase in cyclic guanosine monophosphate, which modulates calcium channels within vascular smooth muscle. Moreover, NO regulates inflammatory responses by suppressing the expression of key pro-inflammatory cytokines, including IL-1β, TNF-α, IL-6, and IFN-γ. Its role extends to the modulation of immune and allergic responses by influencing T-cell proliferation and mast cell secretory activity. Additionally, nitric oxide exhibits antiviral properties through mechanisms such as the inhibition of viral RNA and DNA synthesis [1]. In particular, the role of inhaled nitric oxide (iNO) in the treatment of life-threatening acute respiratory distress syndrome (ARDS) has been thoroughly investigated. Although it has been demonstrated that iNO selectively induces pulmonary vasodilation in ventilated lung areas and improves oxygenation without reducing systemic blood pressure, initially, in small cohort studies [2,3] and later in large randomized controlled trials (RCTs) [4,5,6], no significant impact on mortality has been observed. These findings have been further validated by meta-analyses [7,8,9]. Consequently, there is a recommendation against iNO treatment in both ARDS [5] and Surviving Sepsis Campaign for critically ill COVID-19 patient guidelines [6].

Considering that NO demonstrated antiviral activity against SARS-CoV-1 in the early 2000s and the similarity between this virus and SARS-CoV-2, the potential role of iNO in treating COVID-19 infection has been studied in recent years. Indeed, nitric oxide was found to exhibit virus-suppressing effects in both in vitro and in vivo studies by several possible mechanisms: preventing the virus entering the host–cell, inhibiting viral replication inside the cell, the inactivation of viral particles, and modulating the host immune response [1]. These promising findings, alongside evidence of the efficacy of iNO in ARDS and the profound hypoxemia of COVID-19 patients, led to clinical trials of iNO treatment during the SARS-CoV-2 pandemic and it eventually became a rescue treatment strategy for critically ill coronavirus patients [10].

Several trials in COVID-19 patients have demonstrated that iNO can reduce inflammation, promote viral clearance, and improve clinical outcomes [1].Moreover, systematic reviews have concluded that it has a positive impact on ventilation time and oxygenation in severe COVID-19 patients, with its effect on mortality remaining unclear [11,12].

Studies specifically focused on improving oxygenation with iNO treatment in COVID-19 ventilated patients have examined an immediate (within minutes to hours) and short-term (up to 48 h) improvement [13,14,15,16,17].

Here, we present our experience with inhaled nitric oxide in mechanically ventilated COVID-19 patients, focusing on oxygenation improvement over a follow-up period of up to four days. Additionally, we investigate subgroup-specific responses to iNO, with particular emphasis on gender-specific differences.

## 2. Materials and Methods

### 2.1. Study Design

A retrospective analysis of the electronic database of the tertiary academic Soroka Medical Center in Beer Sheva, Israel, was conducted. The study was approved by the institutional review board (SCRC21049).

### 2.2. Study Population and Data Collection

The study inclusion criteria were as follows: adult patients (≥18 years old) admitted to the medical ICU at Soroka Medical Center between September 2020 and September 2021, fulfilling the Berlin ARDS criteria [18], requiring mechanical protective ventilation due to severe COVID-19 infection that was confirmed by a positive RT-PCR test for SARS-CoV-2 and exhibiting refractory hypoxemia characterized by PaO_2_/FiO_2_ < 150 mm Hg, necessitating treatment with inhaled nitric oxide. The exclusion criteria included pregnancy, ARDS caused by factors other than COVID-19 infection, and the requirement for extracorporeal membrane oxygenation (ECMO) prior to initiating iNO therapy.

The following data were collected: demographics including the Israel Central Bureau of Statistics socio-economic index from 1 (the lowest) to 10 (the highest) [19], medical history, vital signs, blood work results during the admission period, and details of the clinical course of COVID-19 disease such as the need for vasopressors, prone position, and required interventions. Arterial blood gasses and respiratory and ventilation parameters were obtained up to 3 h before and on an hourly basis after each iNO administration. A clinically significant improvement of PaO_2_/FiO_2_ was defined, in accordance with previous studies, as an increase of ≥20% from a baseline [5,20,21].

### 2.3. Mechanical Ventilation and iNO

The patients were sedated and ventilated through an endotracheal or tracheostomy tube according to protective ventilation protocol [22]. The treatment with iNO was started by the decision of the attending senior physician and was given in a continuous way through the NOxBOXi^®^ Nitric Oxide Delivery and Monitoring System (NOXBOX Ltd., Woking, Surrey, UK) with an initial dose of 10 ppm. The dose was titrated up to 20 ppm every hour. The iNO treatment was stopped gradually in case of either oxygenation improvement PaO_2_/FiO_2_ > 150 mm Hg or a decision of end-of-life care protocol initiation.

### 2.4. Statistical Analysis

Descriptive statistics were used to summarize the demographic and clinical characteristics, including mean, median, range, standard deviations (SDs) and interquartile ranges (IQRs).

A longitudinal analysis of the patient-level data was performed to evaluate the impact of iNO administration on the PaO_2_/FiO_2_ ratio. Each participant served as their control, allowing for within-person comparisons of oxygenation levels between periods with and without iNO. This approach eliminated the need for a separate control group, reducing the potential for confounding due to individual-level characteristics.

An interrupted time series approach characterized changes in post-treatment oxygenation levels beyond other clinical variables. This allowed the identification of the proportion of patients, showing a clinically significant improvement in the PaO_2_/FiO_2_ ratio due to iNO administration, along with constructing a confidence interval for this proportion.

The odds of clinical improvement were calculated by comparing the odds ratio from pre to post iNO for each patient using a one-way fixed effects model. This enabled the calculation of odds ratios (ORs), 95% confidence intervals (CIs), and corresponding *p*-values. To identify potentially vulnerable subgroups, the analysis was stratified by sex, age, and the Charlson Comorbidity Index. A subgroup analysis was performed to explore the potential differences in response to iNO across various comorbid conditions. All analyses were performed using R 4.2.2 software.

## 3. Results

One hundred and twelve mechanically ventilated COVID-19 patients who received iNO treatment were identified. Twenty-five patients were excluded due to not fulfilling ARDS criteria or pregnancy. The final study population included 87 patients (55 men and 32 women) with a mean age of 58.7 years (SD ± 15.2). The most common comorbidities were dyslipidemia and diabetes mellitus, affecting 45 (51.7%) and 40 (46%) patients, respectively. Seven (8%) patients were immunocompromised either due to immunosuppressive medication (one on chronic corticosteroid treatment and four with organ transplants) or AIDS (two patients). The patient basic demographics and medical history are presented in Table 1, and the extended data are shown in Supplementary Table S1.

The mean ventilation duration was 7.8 days with mean parameters including a PEEP of 14.4 cmH_2_O, a tidal volume of 512 mL, and a FiO_2_ value of 98.7%. Vasopressor support was required for all but five patients. Fifty patients (57.5%) were placed in a prone position following a connection to iNO with an average duration ranging from 16 to 20 h before transitioning back to the supine position. In thirteen patients (14.9%), the ECMO therapy was initiated, as detailed in Table 2.

Among the 87 study patients, inhaled NO was administered 164 times, resulting in a mean of 1.9 connections per patient, a mean interval of 4.3 days between treatments, and a mean duration of 8.6 days each time. The mean administered dose of iNO was 17.8 ppm (Table 3).

The impact of iNO on respiratory function and acid–base balance at various time intervals over the course of treatment is presented in Table 4. In particular, the PaO_2_/FiO_2_ ratio consistently rose from a baseline of 93.2 to 98.3, 105, 107, 118, and 119 mmHg over 1, 3, 6, 12, and 24 h of treatment, respectively. The number of cases in which this rise was clinically significant (≥20% from the baseline) has also increased over a treatment period from 11 cases (6.7% of all connections) at the first hour of treatment to 74 cases (45.1%) after 24 h. The plateau pressure reduced from 40.7 before iNO to 34.5 mmH_2_O over 24 h of inhalation. The acid–base status changed gradually from mixed respiratory and metabolic acidosis with metabolic alkalosis before the iNO treatment to compensated respiratory acidosis after 24 h of iNO inhalation.

**Figure 1:**
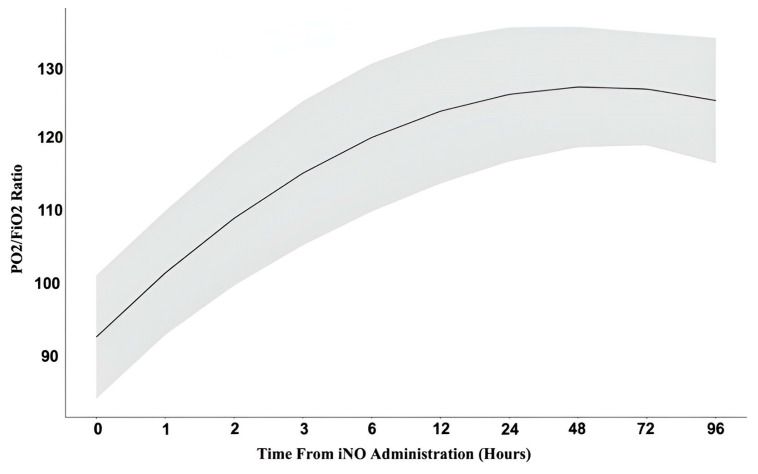
iNO effect on oxygenation up to 96 h. FiO_2_—fraction of inspired oxygen, iNO—inhaled nitric oxide, PO_2_—partial pressure of oxygen in arterial blood. The bold line represents the mean PaO_2_/FiO_2_ ratio; the shaded area depicts the standard deviation values.

**Figure 2:**
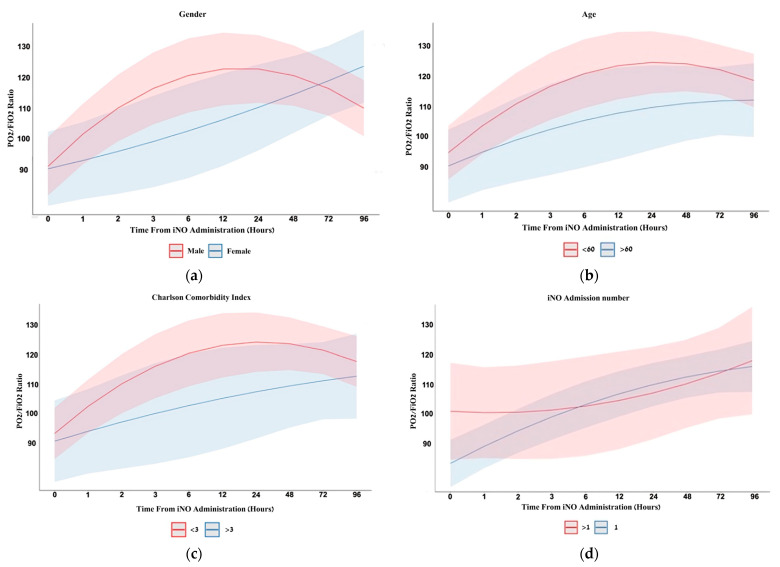
Inhaled NO treatment heterogeneity mixed models. Treatment heterogeneity over time for two groups of patients using a mixed model approach: (**a**) males vs. females, (**b**) age above or under 60 years, (**c**) based on patients’ Charlson comorbidity, (**d**) one or more connections to iNO. FiO_2_—fraction of inspired oxygen, iNO—inhaled nitric oxide, PO_2_—partial pressure of oxygen in arterial blood, PCO_2_—partial pressure of carbon dioxide in arterial blood. The bold line represents the mean PaO_2_/FiO_2_ ratio; the shaded area depicts the standard deviation values.

**Figure 3:**
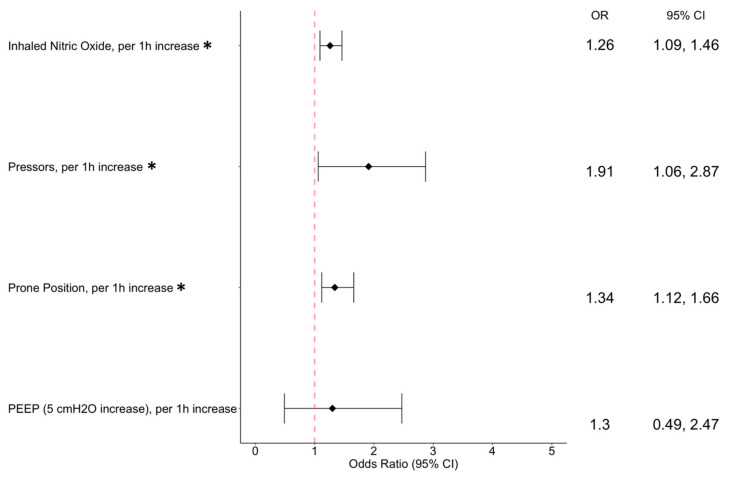
A forest plot of a multivariable analysis regarding the association of various interventions with PaO_2_/FiO_2_ ratio improvement per 1 h of their application. PEEP—positive end-expiratory pressure, iNO—inhaled nitric oxide, *—*p*-value < 0.001.

Figure 1 summarizes the improvement in oxygenation through 96 h of treatment. The iNO effect on the PaO_2_/FiO_2_ ratio was analyzed over time using mixed models to account for heterogeneity (Figure 2). These results were stratified by different demographic and clinical factors (Figure 3), demonstrating that iNO was independently associated with a significant PaO_2_/FiO_2_ improvement, with OR 1.26 (95% CI 1.09–1.46). An analysis of the cumulative impact of multiple comorbidities using the Charlson Comorbidity Index (CCI), along with a subgroup analysis to explore the potential differences in response to iNO across various chronic conditions, was performed (Table 5).

The time to achieve maximal improvement in the PaO_2_/FiO_2_ ratio following iNO initiation was 14.5 ± 5.0 h for men and 78.5 ± 5.5 h for women, with respective ratio increases of 42.5 ± 8.1 and 52.5 ± 13.6 mmHg. No adverse effects of iNO treatment were noticed. Fifty-five patients (63.2%) passed away during their hospital stay.

## 4. Discussion

This study investigates the effect of inhaled nitric oxide in mechanically ventilated ARDS patients due to severe COVID-19 infection.

Our results demonstrate that iNO significantly improves oxygenation in ventilated COVID-19 patients, with a PaO_2_/FiO_2_ increase of 52.5 ± 13.6 mmHg. These findings are consistent with recent studies, including Stoll et al.’s, who reported a PaO_2_/FiO_2_ increase from 138.80 ± 32.50 mmHg to 183.97 ± 49.96 mmHg, and Fenza et al.’s, who observed a mean ratio change of 28.3 mmHg following iNO treatment [13,14,15,16,17]. Furthermore, multivariable analysis reveals that iNO was independently associated with a significant improvement in the PaO_2_/FiO_2_ ratio, showing an OR of 1.26 (95% CI 1.09–1.46), which, even after accounting for these potential confounders, was comparable to other well-known interventions such as prone positioning, with an OR of 1.34 (95% CI 1.12–1.66) (Figure 3).

In comparison to earlier studies, our work has distinct and unique features.

First, whereas previous research has focused on an immediate [23] or short-term oxygenation improvement up to a 48 h [13,14,15,24], our work inquired about the medium-term effect of inhaled NO. We found that the iNO effect is more delayed and sustained than previously reported, persisting up to 96 h post treatment initiation (Figure 1 and Figure 2). This aligns with findings suggesting possible desensitization for inhaled NO after 96 h of treatment in ARDS patients [25]. The clinical significance of our findings is that the absence of immediate improvement within minutes or the first few hours after iNO initiation should not be interpreted as treatment failure. Continued treatment should be considered, as improvement may manifest over several hours or days. This contradicts the recommendations of the previous studies that found no improvement in oxygenation beyond 48 h of iNO treatment [13,16,17] and advocate discontinuing iNO if there is no evidence of a positive effect within the first 24 h [15,26]. The discrepancy can be attributed to variations in the study’s population size (which ranges from dozens to hundreds), the inclusion/exclusion criteria (such as the presence or absence of pulmonary hypertension), and the treatment protocol, including iNO dosing, and whether a prone position was mandatory or optional before iNO initiation.

Second, the pattern of the iNO effect varies across different groups. Patients younger than 60 (Figure 2b) and with fewer comorbidities (Figure 2c, Table 5) may benefit more from this treatment. The subgroup analysis revealed variation in the odds ratios for PaO_2_/FiO_2_ improvement, suggesting that pulmonary hypertension and chronic liver disease may be associated with enhanced response to iNO. The potential impact of pulmonary hypertension on the efficacy of iNO treatment is discussed below in the limitation section. We propose that the effect of chronic liver disease may be explained by impaired NO clearance and increased pulmonary hypertension due to hepato-pulmonary syndrome. In men, the maximum effect is reached within 15 h, whereas in women, the peak effect occurs more gradually, reaching its peak after 78 h (Figure 2a). There is expanding evidence implying that biological sex influences the clinical course and therapeutic responses in various diseases, including COVID-19 infection and outcomes after admission to the intensive care unit. It may be suggested that gender differences in COVID-19 ARDS recovery likely result from a complex interaction of physiological, pharmacokinetic, anatomical, and molecular factors. Specifically, women’s slower metabolism and excretion rates, combined with tighter drug distribution, may delay therapeutic responses. Anatomical differences, such as smaller lung dimensions and fewer alveoli, further impact respiratory recovery. Variations in ACE2 expression, a key receptor for SARS-CoV-2 entry, and differences in immune responses, including reduced estrogen-related protection, may also contribute. While these factors provide valuable insights, the exact mechanisms remain unclear, emphasizing the need for further research to address these disparities [27,28].

Our study has several limitations. Although conducted in a tertiary hospital, it is a single-center study with a potential selection bias. As a retrospective work, it may have observer and recall bias. Additionally, there are limitations in data availability and completeness, such as the inability to directly record plateau pressure and calculate driving pressure and tidal volume indexed to ideal body weight due to the constraints of our electronic medical record system. Although our analysis demonstrated that iNO was independently associated with a significant improvement in PaO_2_/FiO_2_, we acknowledge the inherent limitations of our retrospective design and do not claim causality, as the presence of unmeasured confounders cannot be excluded. Future prospective, randomized studies are essential to establish a definitive causal relationship between iNO and oxygenation improvements in this patient population. Moreover, this study did not aim to assess the impact of iNO treatment on survival outcomes or potential adverse effects such as acute kidney injury related to iNO therapy. This relationship between iNO and clinical outcomes other than oxygenation remains an important area for future investigations. Given the vasodilatory effects of iNO on pulmonary vasculature, it is reasonable to anticipate different responses in patients with pulmonary hypertension compared to those without, as was shown by Garfiled et al. [17]. However, this hypothesis could not be evaluated in our study as routine protocols for critically ill COVID-19 patients at our institution do not include echocardiography or right heart catheterization. Hence, the concern about the potential impact of pulmonary hypertension on the efficacy of iNO treatment in our patients remains unresolved. In addition, our dataset does not include information on the use of muscle relaxants or blood methemoglobin levels, nor does it allow for the analysis of certain subgroup populations.

## 5. Conclusions

Our study provides novel insights into the use of inhaled nitric oxide in severe ARDS COVID-19 patients. Specifically, we demonstrate that the iNO effect of oxygenation improvement is delayed with a prolonged feature that lasts up to 96 h. Furthermore, we identify a gender-based variation in therapeutic response, with women responding slower than men. We suggest that a lack of immediate improvement within minutes to a few hours after iNO initiation should not be seen as treatment failure and iNO initiation should be considered to continue, as benefits may emerge over several hours or days.

Considering the growing evidence supporting iNO as a rescue therapy for patients with severe ARDS in general and COVID-19 in particular, it may be worthwhile to re-examine the current ARDS and Surviving Sepsis Campaign COVID-19 guidelines that currently recommend against its use. In addition, we call for future investigations to explore the underlying mechanisms of these delayed and gender-specific responses, with the goal of developing a more tailored therapeutic approach.

## Figures and Tables

**Table 1:** Baseline characteristics of study patients (n = 87).

Demographics	
Age, years (SD); [range]	58.7 (15.2); [20.7–82.2]
Male gender, n (%)	55 (63.2)
Jewish, n (%)	55 (63.2)
Immigrants, n (%)	31 (35.6)
BMI, kg/m^2^—mean (SD)	33.8 (9.1)
CBS socio-economic index, mean (SD)	4.6 (1.9)
**Comorbidities**	
Dyslipidemia, n (%)	45 (51.7)
Diabetes mellitus, n (%)	40 (46.0)
Atrial fibrillation, n (%)	13 (14.9)
Chronic liver disease, n (%)	12 (13.8)
Chronic kidney disease, n (%)	12 (13.8)
Cerebrovascular disease, n (%)	7 (8.0)
Obstructive lung disease, n (%)	7 (8.0)
Malignant disease, n (%)	6 (6.9)
Pulmonary hypertension, n (%)	5 (5.7)
Organ transplant, n (%)	4 (4.6)
Peripheral vascular disease, n (%)	2 (2.3)
AIDS, n (%)	2 (2.3)
Chronic corticosteroid use, n (%)	1 (1.1)

AIDS—acquired immune deficiency syndrome, BMI—body mass index, CBS—Israel Central Bureau of Statistics, SD—standard deviation.

**Table 2:** Patient ventilation parameters and supportive interventions (n = 87).

Variable	
Prone position, n (%)	50 (57.5)
Vasopressors, n (%)	82 (94.3)
ECMO, n (%)	13 (14.9)
**Ventilation Characteristics**	
Ventilation time, days—mean (SD)	7.8 (8.7)
PEEP, cmH_2_O—mean (SD)	14.4 (7.8)
Max peak pressure, cmH_2_O—mean (SD)	30.6 (10.7)
Respiratory Rate, Insp/min—mean (SD)	27.2 (8.0)
Tidal volume, mL—mean (SD)	512 (99.0)
FiO_2_, %—mean (SD)	98.7 (4.8)
**Ventilation Type ** ^†^	
PCV, n (%)	18 (41.9)
(S)CMV+, n (%)	12 (27.9)
PSV, n (%)	7 (16.3)
ASV, n (%)	4 (9.3)
V/C, n (%)	2 (4.6)

^†^—data from 43 patients, ASV—adaptive support ventilation, ECMO—extracorporeal membrane oxygenation, FiO_2_—fraction of inspired oxygen, PCV—pressure-controlled ventilation, PEEP—positive end-expiratory pressure, PSV—pressure support.

**Table 3:** Characteristics of iNO administration (n = 164) in 87 study patients.

Variable	
Time from admission to iNO use, days—mean (SD)	9.9 (9.7)
Time from ICU admission to iNO use, days—mean (SD)	6.2 (9.0)
Time from COVID-19 diagnosis to iNO use, days—mean (SD)	10.4 (11.4)
Dose, ppm—mean (SD)	17.8 (9.2)
Number of iNO administrations per patient—mean (SD)	1.9 (1.4)
Duration per administration, days—mean (SD)	8.6 (8.7)
Time between administrations, days—mean (SD)	4.3 (8.7)
Total duration of iNO use, days—mean (SD)	16.2 (8.9)

iNO—inhaled nitric oxide, ppm—parts per million, SD—standard deviation.

**Table 4:** The effect of iNO on oxygenation and respiratory function in 164 connections among 87 COVID-19-ARDS patients.

Variable	Before iNO	Over 1 h	Over 3 h	Over 6 h	Over 12 h	Over 24 h
PaO_2_/FiO_2_, mmHg,	93.2	98.3	105	107	118	119
mean (SD)	(52.3)	(76.5)	(59.3)	(58.9)	(68.8)	(61.9)
PaO_2_/FiO_2_ improvement *,		11	13	29	48	74
events (% all connections)		(6.7)	(7.9)	(17.7)	(29.2)	(45.1)
SaO_2_,	90.0	96.2	96.4	97.9	98.2	98.9
%, mean (SD)	(8.4)	(4.8)	(4.5)	(3.4)	(4.0)	(3.6)
A-a gradient,	549	540	535	518	502	488
mmHg, mean (SD)	(79.4)	(53.2)	(79.7)	(98.9)	(102)	(98.9)
Peak flow,	34.9	35.8	36.0	36.7	37.4	38.2
l/min, mean (SD)	(17.5)	(17.4)	(17.6)	(17.4)	(17.2)	(17.1)
Plateau pressure ^†^,	40.7	38.4	37.5	36.4	35.7	34.5
cmH_2_O, mean (SD)	(4.0)	(7.3)	(4.2)	(5.3)	(3.8)	(5.5)
End tidal CO_2_,	31.6	38.3	39.5	39.9	40.2	43.0
mmHg, mean (SD)	(14.2)	(16.7)	(14.9)	(16.4)	(15.8)	(15.5)
pH blood,	7.28	7.30	7.31	7.34	7.36	7.40
mean (SD)	(0.1)	(0.1)	(0.1)	(0.1)	(0.1)	(0.1)
PCO_2_,	65.3	64.1	62.4	61.8	57.6	56.9
mmHg, mean (SD)	(19.7)	(21.4)	(20.6)	(19.1)	(20.0)	(17.4)
HCO_3_,	31.3	29.8	29.0	27.2	26.9	26.4
mEq/L, mean (SD)	(6.5)	(6.9)	(6.7)	(6.3)	(6.0)	(6.9)
Base excess,	7.7	6.0	4.4	3.1	2.8	1.7
mEq/L, mean (SD)	(8.0)	(8.8)	(8.4)	(7.8)	(7.7)	(6.2)
Lactate,	2.7	2.6	2.4	2.4	2.3	2.0
mmol/L, mean (SD)	(3.1)	(2.9)	(2.1)	(1.9)	(2.1)	(1.6)

* PaO_2_/FiO_2_ improvement defined as increase in PaO_2_/FiO_2_ > 20%, ^†^—reported values are approximations of plateau pressure using peak pressure data, FiO_2_—fraction of inspired oxygen, HCO_3_—bicarbonate, iNO—inhaled nitric oxide, PaO_2_—partial pressure of oxygen in arterial blood, PCO_2_—partial pressure of carbon dioxide in arterial blood, SaO_2_—arterial oxygen saturation, SD—standard deviation.

**Table 5:** The subgroup analysis of comorbidities on iNO oxygenation effect among 87 COVID-19-ARDS patients.

Subgroup	Adjusted OR (95% CI)
Dyslipidemia	1.21 (1.05–1.38)
Diabetes Mellitus	1.05 (1.00–1.13)
Atrial Fibrillation	1.07 (0.95–1.18)
Chronic Liver Disease	1.28 (1.12–1.40)
Chronic Kidney Disease	1.03 (0.95–1.08)
Cerebrovascular Disease	1.05 (0.88–1.35)
Obstructive Lung Disease	1.10 (0.95–1.28)
Malignant Disease	1.20 (1.03–1.40)
Pulmonary Hypertension	1.32 (1.20–1.53)
Dyslipidemia	1.21 (1.05–1.38)
BMI < 25 kg/m^2^	1.10 (0.95–1.28)
BMI ≥ 30 kg/m^2^	1.28 (1.05–1.41)

## Data Availability

The data collected during the study and supporting the reported results are not publicly available due to sensitivity concerns. However, they can be obtained from the corresponding author upon reasonable request.

## Supplementary Materials

The following supporting information can be downloaded at: https://www.mdpi.com/article/10.3390/jcm14030806/s1, Table S1: Study patient comorbidities (expanded).
