# Supplementary material for: Medium-Term Effect of Inhaled Nitric Oxide in Mechanically Ventilated COVID-19 Patients
Source: J Clin Med. 2025 Jan 26;14(3):806. doi: 10.3390/jcm14030806 (PMC11818737; doi:10.3390/jcm14030806)
Supplement: Supplementary file 1 [file jcm-14-00806-s001.zip › jcm-3334349-supplementary.pdf]

Suppl. Table S1. Study patient comorbidities and baseline laboratory data (expanded, n = 87).

**Variable**	
Blood count, n (%)	87 (100%)
Hemoglobin, g/dl – mean (SD)	10.6 (2.39)
Leukocytes, K/µl – mean (SD)	16.2 (8.20)
Platelets, K/µl – mean (SD)	252 (111)
Dyslipidemia, n (%)	45 (51.7)
Baseline cholesterol, mg/dl - mean (SD)	178 (36.2)*
Baseline triglycerides, mg/dl - mean (SD)	153 (73.3)*
Baseline HDL, mg/dl - mean (SD)	42.7 (9.9)*
Baseline LDL, mg/dl - mean (SD)	103 (28.8)*
Diabetes mellitus, n (%)	40 (46.0)
Baseline glucose, mg/dl - mean (SD)	170 (70.2)†
Baseline hemoglobin A1C, % - mean (SD)	6.6 (1.0)δ
Atrial fibrillation, n (%)	13 (14.9)
Chronic liver disease, n (%)	12 (13.8)
Baseline ALKP, U/L - mean (SD)	86.0 (45.9)ǂ
Baseline AST, U/L - mean (SD)	40.5 (28.3)†
Baseline ALT, U/L - mean (SD)	36.0 (40.5)‡
Chronic kidney disease, n (%)	12 (13.8)
Baseline creatinine, mg/dl - mean (SD)	1.2 (1.1)†
Baseline urea, mg/dl - mean (SD)	49.2 (32.1)†
Atherosclerosis, n (%)	11 (12.6)
Cerebrovascular disease, n (%)	7 (8.0)
Obstructive lung disease, n (%)	7 (8.0)
Malignant disease, n (%)	6 (6.9)
Pulmonary hypertension, n (%)	5 (5.7)
Organ transplant, n (%)	4 (4.6)
Peripheral vascular disease, n (%)	2 (2.3)
AIDS, n (%)	2 (2.3)
Chronic corticosteroid use, n (%)	1 (1.1)

AIDS - Acquired Immune Deficiency Syndrome, ALKP - alkaline phosphatase, ALT - alanine transaminase, AST - aspartate transaminase, HDL - high-density lipoprotein, LDL - low-density lipoprotein, SD –

standard deviation. $^{\delta}$ - data from 29 patients, * - data from 35 patients, †- data from 60 patients, ‡ - data from 61 patients, ⸸ - data from 62 patients, ⸶- data from 63 patients, ⸷ - data from 64 patients
